# Acute hypoxia impairs posterior cerebral bioenergetics and memory in man

**DOI:** 10.1113/EP091245

**Published:** 2023-10-29

**Authors:** Soichi Ando, Hayato Tsukamoto, Benjamin S. Stacey, Takuro Washio, Thomas S. Owens, Thomas A. Calverley, Lewis Fall, Christopher J. Marley, Angelo Iannetelli, Takeshi Hashimoto, Shigehiko Ogoh, Damian M. Bailey

**Affiliations:** ^1^ Graduate School of Informatics and Engineering The University of Electro‐Communications Tokyo Japan; ^2^ Neurovascular Research Laboratory, Faculty of Life Sciences and Education University of South Wales Pontypridd UK; ^3^ Faculty of Sports Science Waseda University Saitama Japan; ^4^ Department of Biomedical Engineering Toyo University Kawagoe Saitama Japan; ^5^ Faculty of Sport and Health Science Ritsumeikan University Shiga Japan

**Keywords:** cerebral blood flow, cerebral oxygenation, executive function, memory, oxygen delivery

## Abstract

Hypoxia has the potential to impair cognitive function; however, it is still uncertain which cognitive domains are adversely affected. We examined the effects of acute hypoxia (∼7 h) on central executive (Go/No‐Go) and non‐executive (memory) tasks and the extent to which impairment was potentially related to regional cerebral blood flow and oxygen delivery (CDO_2_). Twelve male participants performed cognitive tasks following 0, 2, 4 and 6 h of passive exposure to both normoxia and hypoxia (12% O_2_), in a randomized block cross‐over single‐blinded design. Middle cerebral artery (MCA) and posterior cerebral artery (PCA) blood velocities and corresponding CDO_2_ were determined using bilateral transcranial Doppler ultrasound. In hypoxia, MCA DO_2_ was reduced during the Go/No‐Go task (*P* = 0.010 vs. normoxia, main effect), and PCA DO_2_ was attenuated during memorization (*P* = 0.005 vs. normoxia) and recall components (*P* = 0.002 vs. normoxia) in the memory task. The accuracy of the memory task was also impaired in hypoxia (*P* = 0.049 vs. normoxia). In contrast, hypoxia failed to alter reaction time (*P* = 0.19 vs. normoxia) or accuracy (*P* = 0.20 vs. normoxia) during the Go/No‐Go task, indicating that selective attention and response inhibition were preserved. Hypoxia did not affect cerebral blood flow or corresponding CDO_2_ responses to cognitive activity (*P* > 0.05 vs. normoxia). Collectively, these findings highlight the differential sensitivity of cognitive domains, with memory being selectively vulnerable in hypoxia.

## Abstract (highlights)

- **What is the central question of this study?**
  We sought to examine the effects of acute hypoxia on central executive (selective attention and response inhibition) and non‐executive (memory) performance and the extent to which impairments are potentially related to reductions in regional cerebral blood flow and oxygen delivery.
- **What is the main finding and its importance?**
  Memory was impaired in acute hypoxia, and this was accompanied by a selective reduction in posterior cerebral artery oxygen delivery. In contrast, selective attention and response inhibition remained well preserved. These findings suggest that memory is selectively vulnerable to hypoxia.

## 1 INTRODUCTION

The human brain has evolved to be entirely dependent on O_2_ to support the high rate of ATP formation to fuel the maintenance of ionic equilibria and uptake of neurotransmitters for synaptic transmission (Bailey, 2019a, 2019b). In acute hypoxia, global cerebral blood flow (CBF) increases to maintain cerebral O_2_ delivery (CDO_2_) (Hoiland et al., 2016) and prevent metabolic compromise and/or structural damage to the neurovascular unit (Stacey et al., 2023). Nevertheless, extramitochondrial cellular processes are sensitive to small hypoxic insults, even when CDO_2_ is sufficient to maintain bioenergetic function (Ainslie et al., 2016). Specifically, the synthesis of enzymes and related neurotransmitters is sensitive to O_2_ (Kumar, 2011; Raichle & Hornbein, 2001), and neurotransmitter dysfunction might occur during even subtle reductions in the arterial partial pressure of O_2_ (Ainslie et al., 2016). Consequently, hypoxia appears to have detrimental effects on the CNS and has the potential to impair cognitive function (Ando et al., 2020; McMorris et al., 2017; Taylor et al., 2016; Virues‐Ortega et al., 2004; Wilson et al., 2009).

Cognitive function is classified into central executive and non‐executive domains (Cantelon & Giles, 2021). Central executive function refers to ‘top‐down’ higher cognitive processes (e.g., inhibition, working memory, cognitive flexibility) (Diamond, 2013), whereas non‐executive function includes attention, motor speed, information processing and memory (Cantelon & Giles, 2021). Currently, it is controversial which cognitive domains are most vulnerable to hypoxia (Ando et al., 2020; Petrassi et al., 2012), complicated, in part, by the intrinsic cognitive demands (Ando et al., 2020), considerable participant heterogeneity and experimental failure to account for basal habituation/learning effects (Marley et al., 2017). Conversely, middle temporal lobe‐dependent cognitive function (e.g., memory) is known to be exquisitely vulnerable in hypoxia (Perosa et al., 2020), where structural damage to the hippocampus precedes memory impairment (Zola‐Morgan et al., 1986), which is particularly evident in patients suffering from anoxic brain injury (Garcia‐Molina et al., 2006). Furthermore, breath‐hold diving training over several years can cause mild short‐term memory impairments (Billaut et al., 2018) that might be related to intermittent repetitive structural damage to the neurovascular unit (Bailey et al., 2022). These findings suggest that memory is impaired in hypoxia. However, the underlying mechanisms and associated temporal kinetics remain to be established.

Despite an increase in global CBF in hypoxia, the response is heterogeneous and site specific (Binks et al., 2008; Lawley et al., 2017; Rossetti et al., 2021). Binks et al. (2008) indicated that greater blood flow is directed to phylogenetically older parts of the brain, which suggests that older brain regions might be more bioenergetically demanding to maintain functions in hypoxia compared with newer brain regions (e.g., frontal cortex and central executive function). The hippocampus is phylogenetically old (Murray et al., 2018), and exposure to hypoxia (∼10 h) has been shown to reduce perfusion in the posterior cingulate and cuneal cortex, which are assumed to play a role in declarative and procedural memory (Lawley et al., 2017). Furthermore, maintenance of appropriate hippocampal perfusion is crucial to preserving healthy memory (Johnson, 2023).

Given these knowledge gaps, the purpose of the present study was to examine how acute hypoxia affects central executive and memory functions in a single‐blinded cross‐over design. We hypothesized that (non‐executive) memory would become progressively more impaired in hypoxia, specifically owing to a reduction in CDO_2_ to the posterior circulation, in light of its aforementioned (enhanced) phylogenetic sensitivity.

## 2 MATERIALS AND METHODS

### 2.1 Ethics

Ethical approval for this study was obtained from the Research Ethics Committee at the University of South Wales, UK (#201712BS01). This study conformed to the standards set by the latest revision of the *Declaration of Helsinki*, except for registration in a database, with verbal and written informed consent obtained from all participants.

### 2.2 Participants

Twelve recreationally active males (age, 23 ± 2 years; stature, 1.77 ± 0.07 m; mass, 79 ± 12 kg) were recruited from the University of South Wales via flyers and by word of mouth. All participants lived close to sea level (∼90 m) and had not been exposed to simulated or terrestrial high‐altitude (>2500 m) in the previous 12 months. After a medical examination, they were confirmed to be healthy and free from any known cardiovascular, cerebrovascular or respiratory disease. Furthermore, the participants were not taking any prescribed or over‐the‐counter medications or supplements. They were instructed to refrain from physical activity, caffeine and alcohol and to follow a low‐nitrate/nitrite diet for ≥24 h before the experiment (Bailey et al., 2017). We confirmed that the participants followed these instructions before experimentation via interview.

### 2.3 Design

During the first visit, participants were familiarized with the Go/No‐Go task until their reaction time (RT) was within 2SD from the mean. They were also familiarized with the memory task until they understood the task procedure adequately (see Cognitive function). All participants completed two different experimental conditions in a normobaric environmental chamber (∼120 m^3^) with the ambient temperature maintained at 21°C and relative humidity at 50% (Design Environmental, Ebbw Vale, UK). Participants were randomly exposed to 7 h of either normoxia (fraction of inspired O_2_ = 0.21) or hypoxia (fraction of inspired O_2_ = 0.12) on different visits, separated by ≥7 days. The latter was selected given its established (negative) impact on cognitive function (McMorris et al., 2017). We adopted a block randomization approach using a computer‐based random number generator. Subjects arrived at the laboratory (between 08.00 and 09.00 h) and were fitted with an indwelling cephalic venous cannula. They consumed a standardized meal (30 g of oats with 180 mL water) 30 min before the experimental trials and again at 2, 4 and 6 h to maximize compliance and avoid hunger/dehydration (Ogoh et al., 2021).

**FIGURE 1:**
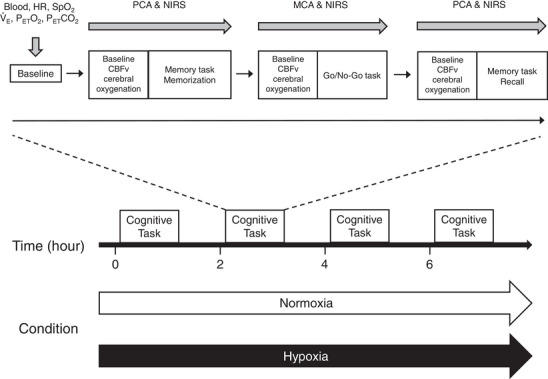
Overview of the experimental protocol. Cognitive function was assessed at baseline (0 h), 2, 4 and 6 h, after measurements of cardiopulmonary and metabolic variables. Abbreviations: CBFv, cerebral blood flow velocity; HR, heart rate; MCA, middle cerebral artery; NIRS, near‐infrared spectroscopy; PCA, posterior cerebral artery; $P_{\mathrm{ET},CO_{2}}$, end‐tidal partial pressures of CO_2_; $P_{\mathrm{ET},O_{2}}$, end‐tidal partial pressures of O_2_; $S_{{\mathrm{pO}}_{2}}$, peripheral O_2_ saturation; $\dot{V}$_E_, minute ventilation.

Cognitive function was assessed at baseline (0 h), 2, 4 and 6 h during each trial (Figure 1). Initially, participants completed baseline measurements of physiological and ventilatory variables. Whole blood was obtained without stasis from an indwelling cannula located in a forearm antecubital vein. The first baseline measurement was performed after passive exposure to hypoxia for 10 min. The other baseline measurements (at 2, 4 and 6 h) were performed after consuming the standardized meal (Figure 1). After baseline measurements, participants performed the cognitive tasks. Before each cognitive task, cerebrovascular variables (baseline CBF velocity and cerebral oxygenation) were measured. This allowed us to assess haemodynamic and O_2_‐delivery responses to cognitive activity. The cognitive tasks started with the memorization part of the memory task, followed by the Go/No‐Go task. Then, the participants performed the recall part of the memory task (Figure 1).

### 2.4 Measurements

#### 2.4.1 Cardiopulmonary

Heart rate (HR) was monitored using a lead II ECG (BioAmp ML132; ADinstruments, UK), and beat‐to‐beat arterial blood pressure was recorded using finger photoplethysmography (Finometer PRO; Finapres Medical Systems, Amsterdam, The Netherlands). Finger photoplethysmography was used to measure beat‐by‐beat systolic blood pressure (SBP), diastolic blood pressure (DBP), stroke volume (SV) and cardiac output ($\dot{Q}$) using the Modelflow algorithm (Wesseling et al., 1993) that incorporates participant sex, age, stature and mass (BeatScope 1.0 software; TNO; TPD Biomedical Instrumentation, Amsterdam, The Netherlands).

Haemoglobin (Hb) was measured photometrically in triplicate (average taken) according to established procedures (B‐Haemoglobin; HemoCue, Sheffield, UK) (Vanzetti, 1966). Peripheral O_2_ saturation ($S_{{\mathrm{pO}}_{2}}$) was measured using a finger‐pulse oximetry (WristOx2 3150, Nonin, MN, USA), and arterial O_2_ content ($C_{{\mathrm{aO}}_{2}}$) was estimated as: [1.39 × Hb × ($S_{{\mathrm{pO}}_{2}}$/100)], where 1.39 is the affinity of O_2_ for Hb. Minute ventilation ($\dot{V}$_E_) and end‐tidal partial pressures of O_2_ and CO_2_ ($P_{\mathrm{ET},O_{2}}$ and $P_{\mathrm{ET},CO_{2}}$) were measured via a mouthpiece and an automatic breath‐by‐breath respiratory gas‐analysis system consisting of a differential pressure transducer, sampling tube, filter, suction pump and mass spectrometer (ML 206; ADInstruments, UK).

#### 2.4.2 Cerebrovascular

Blood velocity in the middle cerebral artery (MCAv; insonated through the left temporal window at a depth of ∼1 cm distal to the MCA–anterior cerebral artery bifurcation) and posterior cerebral artery (PCAv; insonated at the P1 segment through the right temporal window) were measured using standardized procedures (Willie et al., 2011) with a 2 MHz pulsed transcranial Doppler ultrasound (TCD; Spencer Technologies, Seattle, WA, USA). Bilateral TCD probes were secured using a specialized commercial headband (Mark600; Spencer Technologies, Seattle, WA, USA) using standardized search techniques. Between‐day coefficients of variation (CVs) for MCAv and PCAv were 3% and 2%, respectively.

The MCA O_2_/glucose delivery (DO_2_/glucose) and PCA DO_2_/glucose were calculated by multiplying MCAv or PCAv by $C_{{\mathrm{aO}}_{2}}$/plasma glucose concentration. Oxyhaemoglobin (oxy‐Hb) and deoxyhaemoglobin (deoxy‐Hb) were monitored continuously from the left frontal cortex with near‐infrared spectroscopy (NIRS; Oxymon Mk III; Artinis Medical Systems, Zetten, The Netherlands) (Brugniaux et al., 2014). The Oxy‐Hb and deoxy‐Hb were expressed relative to changes from baseline, arbitrarily defined as 0 μmol/L. The tissue saturation index (TSI) was expressed as [oxy‐Hb/(oxy‐Hb + deoxy‐Hb)] × 100 (as a percentage).

#### 2.4.3 Metabolic

Blood was collected into 1 mL syringes to determine Hb and into vacutainers containing EDTA‐2Na for plasma samples. Each vacutainer was gently mixed and centrifuged at 600*g* (4°C) for 10 min. Plasma supernatant was stored at −80°C for glucose and lactate analysis using a Randox Daytona‐plus Clinical Chemistry analyser (Prior et al., 2020). For glucose measurement, there was an analytical sensitivity of 0.02 mmol/L and a dynamic range of 0.02–250 mmol/L, with an intra‐assay CV of <7.1% (Prior et al., 2020). For lactate measurement, there was an analytical sensitivity of 0.5 mmol/L and a dynamic range of 0.5−40 mmol/L, with an intra‐assay CV of <1.5%. Insulin was measured using an Invitron Insulin ELISA kit, with an analytical sensitivity of 0.02 mU/L and a dynamic range of 0.02−250 mU/L, with an inter‐assay CV of ≤ 7.1% (Prior et al., 2020).

### 2.5 Cognitive function

The cognitive task comprised central executive function (Go/No‐Go) and a non‐executive (memory) task (Akagi et al., 2019; Saito et al., 2021; Washio et al., 2021). The Go/No‐Go task evaluates selective attention and response inhibition (Ando et al., 2013), and the memory task requires non‐executive recall memory (Akagi et al., 2019; Lefferts et al., 2016a). The cognitive tasks were programmed and controlled using the software Presentation (v.19; NeuroBehavioral Systems, Berkeley, CA, USA). The participants viewed a computer screen placed ∼80 cm away from them in a supine position. The order of the cognitive tasks is shown in Figure 1. In the memory task, 30 words were presented to the participants for memorization and later recall from memory. The list contained 30 concrete English words that were displayed for 1 s each. We used different word sets for each measurement in the memory task to avoid learning effects. The participants then completed the Go/No‐Go task before the recall part of the memory task. In the recall part, 60 words (including 30 distracters) were presented, at a rate of one word every 2 s. In the Go/No‐Go task, each trial started with a blank screen for 2.5 s, followed by a green square presentation at the centre of the computer screen for 1 s, which served as a preparatory stimulus. Then, one of four squares (red, blue, yellow and purple) was presented for 1 s. Half of the participants responded to red and blue squares as Go signals, and the others responded to yellow and purple squares as Go signals. In the Go trial, the participants were instructed to press a mouse button with the right index finger as quickly as possible. For the No‐Go trial, the participants were instructed to withhold their response. The Go/No‐Go task consisted of a block of 60 trials with equal probability. In the recall part of the memory task, the participants were instructed to press the mouse button if the word had been presented during the memorization.

Performance of the Go/No‐Go task was assessed by RT and accuracy (as a percentage). Omissions of a response in a Go‐trial or incorrect response in a No‐Go trial were regarded as an error. Memory function was assessed by accuracy (as a percentage). All accuracy calculations were determined as the number of correct responses/total number of trials (×100).

### 2.6 Data sampling

Beat‐by‐beat data were sampled continuously at 1 kHz using an analog‐to‐digital converter (Powerlab/16SP ML795; ADInstruments, Colorado Springs, CO, USA) stored on a personal computer for off‐line analysis (LabChart v.7.2.2, ADInstruments, Colorado Springs, CO, USA). All files were given a coded number (not named) by an investigator blinded to the study. We also time‐aligned the mean arterial blood pressure (MAP) and TCD channels, given the time delay (1.07 s) associated with MAP signal processing when using the Finometer PRO (Bailey et al., 2013). Baseline physiological and ventilatory variables were averaged over 1 min. Cerebrovascular variables (i.e., MCAv, PCAv, oxy‐Hb, deoxy‐Hb and TSI) were averaged over 30 s at baseline. Cerebrovascular variables were also averaged during the cognitive tasks.

### 2.7 Statistical analysis

Data were analysed using SPSS v.29.0 (SPSS, Chicago, IL, USA). Distribution normality was assessed using repeated Shapiro–Wilk *W* tests. We performed a two‐way repeated‐measures ANOVA [condition (normoxia vs. hypoxia) × time (0, 2, 4 and 6 h)] for physiological, ventilatory and biochemical variables. We also performed a three‐way repeated‐measures ANOVA [condition (normoxia vs. hypoxia) × cognitive activity (baseline vs. cognitive task) × time (0, 2, 4 and 6 h)] for MCAv and PCAv and corresponding CDO_2_/glucose. For Δoxy‐Hb, Δdeoxy‐Hb and ΔTSI, a three‐way ANOVA [cognitive task (memorization part, Go/No‐Go task and recall part) × condition (normoxia vs. hypoxia) × time (0, 2, 4 and 6 h)] was performed. The ANOVAs were followed by Bonferroni multiple comparisons/paired t‐tests for normally distributed data or the Wilcoxon signed rank test for non‐normally distributed data. Bonferroni correction was applied to correct for multiple testing where appropriate. The degree of freedom was corrected using the Huynh Feldt Epsilon when the assumption of sphericity was violated. Retrospective effect sizes are presented as partial eta‐squared (η_p_^2^). Data are expressed as the mean (SD) or median (interquartile range), and the significance level was set at *P* < 0.05 for all two‐tailed tests.

## 3 RESULTS

### 3.1 Basal responses

Baseline physiological and metabolic variables are summarized in Table 1. Hypoxia increased HR (*P* = 0.009 vs. normoxia, main effect), $\dot{Q}$ (*P* = 0.014 vs. normoxia) and lactate (*P* = 0.046 vs. normoxia). Hypoxia decreased $S_{{\mathrm{pO}}_{2}}$, $C_{{\mathrm{aO}}_{2}}$, $P_{\mathrm{ET},O_{2}}$ and $P_{\mathrm{ET},CO_{2}}$ (*P* < 0.001 vs. normoxia). Heart rate, $\dot{Q}$, $S_{{\mathrm{pO}}_{2}}$, $C_{{\mathrm{aO}}_{2}}$, $\dot{V}$_E_, $P_{\mathrm{ET},CO_{2}}$ and lactate changed as a function of time (*P* < 0.05, main effect), and interaction effects were also apparent for these variables except for $P_{\mathrm{ET},CO_{2}}$ (*P* < 0.05). In hypoxia, HR and $\dot{Q}$ increased at 6 h compared with 0 h (HR, *P* = 0.003; $\dot{Q}$, *P* = 0.007). Increases in $S_{{\mathrm{pO}}_{2}}$ (*P* = 0.036) and lactate (*P* = 0.010) were observed at 4 h (vs. 0 h). $C_{{\mathrm{aO}}_{2}}$ increased at 4 h compared with 0 h (*P* = 0.002) and 2 h (*P* = 0.010) and remained elevated at 6 h (*P* = 0.022 vs. 0 h). $\dot{V}$_E_ increased at 4 h (*P* < 0.001 vs. 0 h) and 6 h (*P* < 0.001 vs. 0 h; *P* < 0.001 vs. 2 h), resulting in a corresponding reduction in $P_{\mathrm{ET},CO_{2}}$ at 4 h (*P* = 0.001 vs. 0 h) and 6 h (*P* < 0.001 vs. 0 h; *P* = 0.002 vs. 2 h).

**TABLE 1:** Basal cognitive, physiological and cerebrovascular responses to hypoxia.

Variable	Condition	0 h	2 h	4 h	6 h	Main effect		Interaction
						Condition	Time	
Accuracy of the Go/No‐Go task (%)	Normoxia	100 (98.7–100)	100 (98.3–100)	98.3 (97.1–100)	100 (98.3–100)	*F*_1,11_ = 1.846, *P* = 0.201 η_p_^2^ = 0.144	*F*_3,33_ = 0.956, *P* = 0.425 η_p_^2^ = 0.080	*F*_3,33_ = 0.431, *P* = 0.732 η_p_^2^ = 0.038
	Hypoxia	98.3 (93.0–100)	98.3 (96.7–100)	97.5 (95.4–98.3)	98.3 (97.1–100)			
HR (beats/min)	Normoxia	57 ± 12	59 ± 10	58 ± 10	56 ± 8	*F*_1,10_ = 10.286, ***P* = 0.009** η_p_^2^ = 0.507	*F*_3,30_ = 3.684, ***P* = 0.023** η_p_^2^ = 0.269	*F*_3,30_ = 4.387, ***P* = 0.011** η_p_^2^ = 0.305
	Hypoxia	61 ± 10	65 ± 14	66 ± 13	73 ± 13^††^			
SBP (mmHg)	Normoxia	140 ± 15	144 ± 13	136 ± 22	149 ± 12	*F*_1,6_ = 1.389, *P* = 0.283 η_p_^2^ = 0.188	*F*_3,18_ = 1.368, *P* = 0.285 η_p_^2^ = 0.186	*F*_3,18_ = 1.665, *P* = 0.210 η_p_^2^ = 0.217
	Hypoxia	137 ± 8	140 ± 24	127 ± 13	129 ± 9			
DBP (mmHg)	Normoxia	67 ± 7	61 ± 8	63 ± 10	66 ± 12	*F*_1,6_ = 3.569, *P* = 0.108 η_p_^2^ = 0.373	*F*_3,18_ = 0.530, *P* = 0.668 η_p_^2^ = 0.081	*F*_3,18_ = 0.465, *P* = 0.710 η_p_^2^ = 0.072
	Hypoxia	58 ± 12	58 ± 14	53 ± 7	58 ± 8			
MAP (mmHg)	Normoxia	92 ± 9	89 ± 6	88 ± 12	94 ± 11	*F*_1,6_ = 3.260, *P* = 0.121 η_p_^2^ = 0.352	*F*_3,18_ = 0.784, *P* = 0.519 η_p_^2^ = 0.116	*F*_3,18_ = 1.007, *P* = 0.412 η_p_^2^ = 0.144
	Hypoxia	85 ± 10	84 ± 14	78 ± 8	80 ± 7			
*Q̇* (L/min)	Normoxia	5.7 ± 1.2	5.9 ± 1.0	5.9 ± 1.0	5.7 ± 0.8	*F*_1,9_ = 9.325, ***P* = 0.014** η_p_^2^ = 0.509	*F*_3,27_ = 2.982, ***P* = 0.049** η_p_^2^ = 0.249	*F*_3,27_ = 3.711, ***P* = 0.023** η_p_^2^ = 0.292
	Hypoxia	6.2 ± 1.0	6.6 ± 1.4	6.7 ± 1.3	7.3 ± 1.3^††^			
$S_{{\mathrm{pO}}_{2}}$ (%)	Normoxia	99 (98–99)	98 (97–99)	98 (97–98)	98 (98–98)	*F*_1,11_ = 355.085, ***P* < 0.001** η_p_^2^ = 0.970	*F*_3,33_ = 3.344, ***P* = 0.031** η_p_^2^ = 0.233	*F*_3,33_ = 4.140, ***P* = 0.013** η_p_^2^ = 0.273
	Hypoxia	84 ± 5	83 ± 4	87 ± 3^†^	86 ± 4			
$C_{{\mathrm{aO}}_{2}}$ (mL/dL)	Normoxia	20.0 ± 1.7	20.5 ± 1.9	20.4 ± 1.4	20.2 (19.9–20.7)	*F*_1,11_ = 76.992, ***P* < 0.001** η_p_^2^ = 0.875	*F*_3,33_ = 6.057, ***P* = 0.002** η_p_^2^ = 0.355	*F*_3,33_ = 3.583, ***P* = 0.024** η_p_^2^ = 0.246
	Hypoxia	16.4 ± 1.5	16.6 ± 1.3	17.7 ± 1.4^††‡^	17.4 ± 1.3^†^			
*V̇*_E_ (L/min)	Normoxia	12.7 (11.8–15.6)	15.5 ± 4.6	15.5 ± 4.8	16.1 ± 4.4	*F*_1,9_ = 0.763, *P* = 0.405 η_p_^2^ = 0.078	*F*_3,27_ = 22.053, ***P* < 0.001** η_p_^2^ = 0.710	*F*_3,27_ = 3.682, ***P* = 0.024** η_p_^2^ = 0.290
	Hypoxia	14.3 ± 2.1	15.8 ± 3.2	17.6 ± 13.2^†††^	19.3 ± 2.8^†††‡‡‡^			
$P_{\mathrm{ET},O_{2}}$ (mmHg)	Normoxia	93 ± 5	92 ± 5	92 ± 3	93 ± 4	*F*_1,9_ = 1195.057, ***P* < 0.001**η_p_^2^ = 0.993	*F*_2.206,19.850_ = 1.297, *P* = 0.298η_p_^2^ = 0.126	*F*_3,27_ = 1.463, *P* = 0.247 η_p_^2^ = 0.140
	Hypoxia	40 (38–42)	43 ± 4	44 ± 4	46 ± 6			
$P_{\mathrm{ET},CO_{2}}$ (mmHg)	Normoxia	43 ± 6	43 ± 4	43 ± 4	43 ± 4	*F*_1,9_ = 31.648, ***P* < 0.001** η_p_^2^ = 0.779	*F*_3,27_ = 8.311, ***P* < 0.001** η_p_^2^ = 0.480	*F*_1.464,13.177_ = 4.122, *P* = 0.051 η_p_^2^ = 0.314
	Hypoxia	38 ± 3	35 ± 4	33 ± 4^††^	32 ± 4^†††‡‡^			
Glucose (mmol/L)	Normoxia	6.1 ± 1.0	6.2 ± 0.9	5.9 (5.6–6.9)	6.2 ± 1.0	*F*_1,9_ = 1.716, *P* = 0.223 η_p_^2^ = 0.160	*F*_3,27_ = 1.331, *P* = 0.285 η_p_^2^ = 0.129	*F*_3,27_ = 1.369, *P* = 0.274 η_p_^2^ = 0.132
	Hypoxia	6.1 (5.9–6.9)	5.6 (5.2–6.2)	6.8 ± 0.9	6.4 ± 0.7			
Insulin (pmol/L)	Normoxia	60 ± 39	64 ± 57	77 ± 62	42 (22–89)	*F*_1,9_ = 0.003, *P* = 0.959 η_p_^2^ = 0.000	*F*_3,27_ = 0.883, *P* = 0.462 η_p_^2^ = 0.089	*F*_2.024,18.213_ = 0.176, *P* = 0.842 η_p_^2^ = 0.019
	Hypoxia	55 (41–78)	52 ± 44	41 (24–149)	35 (22–102)			
Lactate (mmol/L)	Normoxia	0.9 ± 0.1	0.9 ± 0.2	0.9 ± 0.1	0.8 ± 0.2	*F*_1,9_ = 5.369, ***P* = 0.046** η_p_^2^ = 0.374	*F*_3,27_ = 3.858, ***P* = 0.020** η_p_^2^ = 0.300	*F*_1.849,16.641_ = 3.898, ***P* = 0.044**η_p_^2^ = 0.302
	Hypoxia	0.9 ± 0.2	1.1 ± 0.3	1.2 ± 0.5^†^	1.2 ± 0.4			

*Note*: Values are the mean ± SD or median (interquartile range). Bold font indicates statistically significant.

Abbreviations: $C_{{\mathrm{aO}}_{2}}$, arterial O_2_ content; DBP, diastolic blood pressure; HR, heart rate; MAP, mean arterial blood pressure; $\dot{Q}$, cardiac output; $P_{\mathrm{ET},CO_{2}}$, end‐tidal partial pressure of CO_2_; $P_{\mathrm{ET},O_{2}}$, end‐tidal partial pressures of O_2_; SBP, systolic blood pressure; $S_{{\mathrm{pO}}_{2}}$, peripheral O_2_ saturation; $\dot{V}$_E_, minute ventilation.

^†^ *P* < 0.05, ^††^ *P* < 0.01, ^†††^ *P* < 0.001 vs. 0 h. ^‡^ *P* < 0.05, ^‡‡^ *P* < 0.01, ^‡‡‡^ *P* < 0.001 vs. 2 h.

### 3.2 Haemodynamic responses to cognitive activity

**FIGURE 2:**
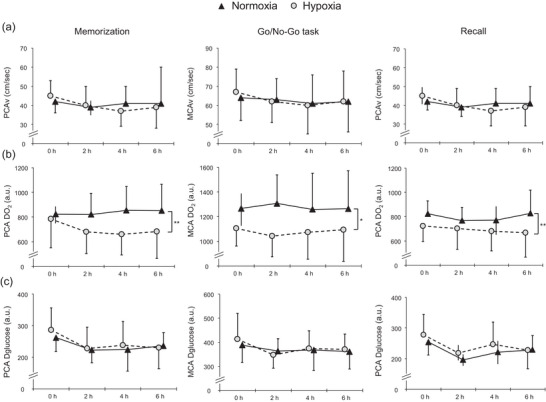
The PCAv/MCAv (a), PCA/MCA DO_2_ (b) and PCA/MCA Dglucose (c) during memorization in the memory task, the Go/No‐Go task and the recall part in the memory task. Black triangles represent normoxia. Grey circles represent hypoxia. Values are the mean ± SD or median (interquartile range). Asterisks highlight main effects for condition (see Table 2). ^*^ *P* < 0.05, ^**^ *P* < 0.01. Abbreviations: Dglucose, glucose delivery; DO_2_, O_2_ delivery; MCAv, middle cerebral artery velocity; PCAv, posterior cerebral artery velocity.

Cognitive activity did not affect PCAv/MCAv and PCA/MCA DO_2_/glucose (*P* > 0.05, main effect; Table 2). The PCAv/MCAv and PCA/MCA DO_2_/glucose during the cognitive tasks are illustrated in Figure 2. Hypoxia reduced PCA DO_2_ during the memorization part (*P* = 0.005 vs. normoxia, main effect), MCA DO_2_ during the Go/No‐Go task (*P* = 0.010 vs. normoxia) and PCA DO_2_ during the recall part (*P* = 0.002 vs. normoxia). Hypoxia did not affect CBF and CDglucose (*P* > 0.05 vs. normoxia).

**TABLE 2:** Cerebral bioenergetic responses to cognitive activity.

Variable	Condition	Timing of measurement	0 h	2 h	4 h	6 h	Main effect		
							Condition	Cognitive activity	Time
Memorization part of the memory task									
PCAv (cm/s)	Normoxia	Baseline	42 ± 5	41 ± 9	42 ± 10	42 ± 9	*F*_1,8_ = 0.344 *P* = 0.574 η_p_^2^ = 0.041	*F*_1,8_ = 0.168 *P* = 0.693 η_p_^2^ = 0.021	*F*_3,24_ = 2.524 *P* = 0.082 η_p_^2^ = 0.240
		During cognitive task	42 ± 6	39 (34–41)	41 ± 9	41 ± 10			
	Hypoxia	Baseline	44 ± 8	39 ± 8	37 ± 9	38 ± 9			
		During cognitive task	45 ± 8	40 ± 10	37 ± 8	39 ± 11			
PCA DO_2_ (a.u.)	Normoxia	Baseline	843 ± 109	850 ± 192	865 ± 210	861 ± 190	*F*_1,8_ = 14.646 ***P* = 0.005** η_p_^2^ = 0.647	*F*_1,8_ = 0.068 *P* = 0.802 η_p_^2^ = 0.008	*F*_2.439,19.515_ = 0.639 *P* = 0.568 η_p_^2^ = 0.074
		During cognitive task	823 (770–907)	820 ± 171	854 ± 194	851 ± 213			
	Hypoxia	Baseline	728 ± 144	655 ± 160	672 ± 186	668 ± 190			
		During cognitive task	784 (631–864)	681 ± 177	660 ± 168	683 ± 220			
PCA Dglucose (a.u.)	Normoxia	Baseline	261 ± 44	210 (192–241)	243 ± 46	242 ± 45	*F*_1,6_ = 0.011 *P* = 0.919 η_p_^2^ = 0.002	*F*_1,6_ = 0.032 *P* = 0.863 η_p_^2^ = 0.005	*F*_3,18_ = 2.176 *P* = 0.126 η_p_^2^ = 0.266
		During cognitive task	262 ± 44	223 ± 40	224 (214–282)	236 ± 42			
	Hypoxia	Baseline	284 ± 80	204 (185–229)	238 ± 75	230 ± 60			
		During cognitive task	286 ± 70	228 ± 67	238 ± 75	230 ± 66			
Go/No‐Go task									
MCAv (cm/s)	Normoxia	Baseline	65 ± 14	62 ± 12	61 ± 14	62 ± 16	*F*_1,8_ = 0.315 *P* = 0.590 η_p_^2^ = 0.038	*F*_1,8_ = 2.465 *P* = 0.155 η_p_^2^ = 0.236	*F*_3,24_ = 2.919 *P* = 0.055 η_p_^2^ = 0.267
		During cognitive task	64 ± 12	63 ± 11	61 ± 15	62 ± 16			
	Hypoxia	Baseline	69 ± 17	63 ± 10	61 ± 12	65 ± 12			
		During cognitive task	67 ± 11	62 ± 10	60 ± 13	62 ± 12			
MCA DO_2_ (a.u.)	Normoxia	Baseline	1304 ± 260	1294 ± 244	1252 ± 271	1267 ± 326	*F*_1,8_ = 11.111 ***P* = 0.010** η_p_^2^ = 0.581	*F*_1,8_ = 2.017 *P* = 0.193 η_p_^2^ = 0.201	*F*_3,24_ = 0.512 *P* = 0.678 η_p_^2^ = 0.060
		During cognitive task	1266 (1114–1374)	1306 ± 231	1258 ± 294	1264 ± 308			
	Hypoxia	Baseline	1129 ± 226	1068 ± 179	1085 ± 243	1140 ± 250			
		During cognitive task	1105 ± 143	1042 ± 168	1074 ± 219	1093 ± 255			
MCA Dglucose (a.u.)	Normoxia	Baseline	400 ± 96	361 ± 48	368 ± 85	348 ± 57	*F*_1,6_ = 0.497 *P* = 0.507 η_p_^2^ = 0.076	*F*_1,6_ = 1.297 *P* = 0.298 η_p_^2^ = 0.178	*F*_3,18_ = 1.142 *P* = 0.359 η_p_^2^ = 0.160
		During cognitive task	388 ± 72	363 ± 52	368 ± 85	361 ± 72			
	Hypoxia	Baseline	419 ± 150	360 ± 51	380 ± 73	384 ± 54			
		During cognitive task	413 ± 107	347 ± 55	374 ± 74	370 ± 64			
Recall part of the memory task									
PCAv (cm/s)	Normoxia	Baseline	41 ± 5	39 ± 8	39 ± 8	40 ± 9	*F*_1,8_ = 0.157 *P* = 0.702 η_p_^2^ = 0.019	*F*_1,8_ = 0.101 *P* = 0.759 η_p_^2^ = 0.012	*F*_3,24_ = 1.676 *P* = 0.199 η_p_^2^ = 0.173
		During cognitive task	41 ± 5	37 ± 5	40 ± 8	40 ± 9			
	Hypoxia	Baseline	43 ± 8	42 ± 10	37 ± 8	38 ± 10			
		During cognitive task	44 (41–50)	42 ± 9	38 ± 8	38 ± 10			
PCA DO_2_ (a.u.)	Normoxia	Baseline	825 ± 107	810 ± 164	817 ± 168	819 ± 175	*F*_1,8_ = 19.155 ***P* = 0.002** η_p_^2^ = 0.705	*F*_1,8_ = 0.061 *P* = 0.812 η_p_^2^ = 0.008	*F*_1.883,15.067_ = 0.267 *P* = 0.756 η_p_^2^ = 0.032
		During cognitive task	824 ± 106	767 ± 110	771 (709–936)	827 ± 192			
	Hypoxia	Baseline	707 ± 149	700 ± 193	662 ± 153	665 ± 198			
		During cognitive task	722 ± 128	703 ± 173	681 ± 163	667 ± 200			
PCA Dglucose (a.u.)	Normoxia	Baseline	256 ± 47	218 ± 42	235 ± 47	229 ± 42	*F*_1,6_ = 0.968 *P* = 0.363 η_p_^2^ = 0.139	*F*_1,6_ = 0.037 *P* = 0.855 η_p_^2^ = 0.006	*F*_3,18_ = 2.024 *P* = 0.147 η_p_^2^ = 0.252
		During cognitive task	254 ± 42	196 (190–227)	221 (211–286)	229 ± 46			
	Hypoxia	Baseline	274 ± 70	240 ± 71	243 ± 77	231 ± 72			
		During cognitive task	278 ± 66	218 (199‐249)	247 ± 72	228 ± 61			

*Note*: Values are the mean ± SD or median (interquartile range). Bold font indicates statistically significant.

Abbreviations: MCA Dglucose, middle cerebral artery glucose delivery; MCA DO_2_, middle cerebral artery O_2_ delivery; MCAv, middle cerebral artery blood velocity; PCA Dglucose, posterior cerebral artery glucose delivery; PCA DO_2_, posterior cerebral artery O_2_ delivery; PCAv, posterior cerebral artery blood velocity.

**FIGURE 3:**
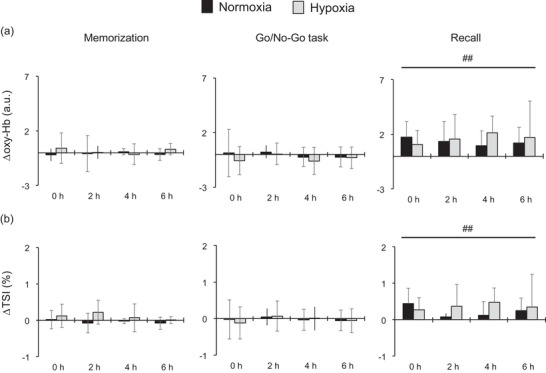
The ΔOxy‐Hb (a) and ΔTSI (b) during memorization in the memory task, the Go/No‐Go task and the recall part in the memory task. Values are the mean ± SD or median (interquartile range). ^##^ *P* < 0.01 vs. memorization and Go/No‐Go task. Abbreviations: oxy‐Hb, oxyhaemoglobin; TSI, tissue saturation index. Numbers represent the time (in hours) after the exposure to hypoxia/normoxia.

Cerebral oxygenation responses to cognitive activity are illustrated in Table 3. We observed that oxy‐Hb and TSI changes in response to cognitive tasks were different between the memorization part, the Go/No‐Go task and the recall part (*P* < 0.001, main effects). In contrast, deoxy‐Hb change was not different (*P* = 0.435, main effect). Figure 3 shows Δoxy‐Hb (Figure 3a) and ΔTSI (Figure 3b) in response to cognitive activity. Hypoxia did not affect Δoxy‐Hb and ΔTSI (*P* > 0.05, main effects). Both Δoxy‐Hb and ΔTSI were greater during the recall part compared with memorization and the Go/No‐Go task (Δoxy‐Hb: *P* = 0.002 vs. memorization, *P* < 0.001 vs. Go/No‐Go task; ΔTSI: *P* = 0.009 vs. memorization, *P* = 0.001 vs. Go/No‐Go task).

**TABLE 3:** Cerebral oxygenation responses to cognitive activity.

						Main effect		
Variable	Condition	0 h	2 h	4 h	6 h	Cognitive task	Condition	Time
Memorization part of the memory task								
ΔOxy‐Hb (μmol/L)	Normoxia	−0.2 (−0.9, 0.9)	−0.3 ± 1.7	0.1 ± 0.3	−0.1 ± 0.6	*F*_2,18_ = 15.328 ***P* < 0.001**, η_p_^2^ = 0.630	*F*_1,9_ = 0.040 *P* = 0.846, η_p_^2^ = 0.004	*F*_3,27_ = 0.575 *P* = 0.636, η_p_^2^ = 0.060
	Hypoxia	0.4 ± 1.5	0.0 (−0.4, 1.4)	−0.2 ± 1.0	0.4 ± 0.6			
ΔDeoxy‐Hb (μmol/L)	Normoxia	−0.1 (−0.4, 0.3)	0.1 ± 0.9	0.2 ± 0.2	0.3 (0.0, 0.5)	*F*_2,18_ = 0.872 *P* = 0.435, η_p_^2^ = 0.088	*F*_1,9_ = 0.731 *P* = 0.415, η_p_^2^ = 0.075	*F*_3,27_ = 0.178 *P* = 0.910, η_p_^2^ = 0.019
	Hypoxia	−0.1 ± 0.5	−0.3 ± 0.8	−0.5 ± 1.1	0.4 ± 0.8			
ΔTSI (%)	Normoxia	0.0 ± 0.3	−0.1 ± 0.3	0.0 ± 0.1	−0.1 ± 0.2	*F*_2,18_ = 13.803 ***P* < 0.001**, η_p_^2^ = 0.605	*F*_1,9_ = 0.317 *P* = 0.587, η_p_^2^ = 0.034	*F*_3,27_ = 0.407 *P* = 0.749, η_p_^2^ = 0.043
	Hypoxia	0.1 ± 0.4	0.2 ± 0.4	0.1 ± 0.4	0.0 ± 0.1			
Go/No‐Go task						Interaction		
ΔOxy‐Hb (μmol/L)	Normoxia	0.1 ± 2.2	0.2 (−0.2, 1.3)	−0.2 ± 0.9	−0.2 ± 0.9	Cognitive task × condition	Δoxy‐Hb: *F*_2,18_ = 1.893, *P* = 0.179, η_p_^2^ = 0.174	
	Hypoxia	−0.6 ± 1.3	0.0 ± 1.0	−0.6 ± 1.2	−0.3 ± 1.0	Δdeoxy‐Hb: *F*_2,18_ = 0.956, *P* = 0.399, η_p_^2^ = 0.096		
ΔDeoxy‐Hb (μmol/L)	Normoxia	0.2 (−0.5, 0.4)	0.3 ± 0.6	−0.1 ± 0.7	0.0 ± 0.4	ΔTSI: *F*_2,18_ = 2.160, *P* = 0.144, η_p_^2^ = 0.194		
	Hypoxia	−0.1 ± 1.2	−0.2 ± 1.1	−0.1 (−0.4, 0.5)	−0.1 ± 1.1	Cognitive task × time	Δoxy‐Hb: *F*_6,54_ = 0.707, *P* = 0.645, η_p_^2^ = 0.073	
ΔTSI (%)	Normoxia	0.0 ± 0.5	0.0 (−0.3, 0.3)	0.0 ± 0.3	0.0 ± 0.3	Δdeoxy‐Hb: *F*_6,54_ = 1.230, *P* = 0.306, η_p_^2^ = 0.120		
	Hypoxia	−0.1 ± 0.4	0.1 ± 0.4	0.0 (−0.3, 0.1)	−0.1 ± 0.3	ΔTSI: *F*_6,54_ = 1.050, *P* = 0.404, η_p_^2^ = 0.105		
						Condition × time	Δoxy‐Hb: *F*_3,27_ = 1.250, *P* = 0.311, η_p_^2^ = 0.122	
Recall part of the memory task						Δdeoxy‐Hb: *F*_3,27_ = 0.972, *P* = 0.420, η_p_^2^ = 0.097		
ΔOxy‐Hb (μmol/L)	Normoxia	1.9 ± 1.4	1.3 ± 1.8	0.8 ± 1.4	1.3 ± 1.5	ΔTSI: F_3,27_ = 0.540, *P* = 0.659, η_p_^2^ = 0.057		
	Hypoxia	0.7 ± 1.3	1.0 ± 2.3	2.0 ± 1.5	2.0 ± 3.3	Cognitive task × condition × time	Δoxy‐Hb: *F*_6,54_ = 0.697, *P* = 0.653, η_p_^2^ = 0.072	
ΔDeoxy‐Hb (μmol/L)	Normoxia	−0.1 ± 0.7	0.4 (0.2, 0.8)	0.4 ± 0.8	0.3 (−0.4, 0.6)	Δdeoxy‐Hb: *F*_6,54_ = 1.085, *P* = 0.383, η_p_^2^ = 0.108		
	Hypoxia	−0.4 (−0.5, 0.4)	0.2 ± 0.8	0.1 ± 1.8	0.0 ± 0.7	ΔTSI: *F*_6,54_ = 0.849, *P* = 0.538, η_p_^2^ = 0.086		
ΔTSI (%)	Normoxia	0.5 ± 0.4	0.1 (0.0, 0.2)	0.1 ± 0.4	0.3 ± 0.3			
	Hypoxia	0.2 ± 0.3	0.2 ± 0.6	0.5 ± 0.4	0.4 ± 0.9			

*Note*: Values are the mean ± SD or median (interquartile range). Bold font indicates statistically significant.

Abbreviations: Deoxy‐Hb, deoxyhaemoglobin; Oxy‐Hb, oxyhaemoglobin; TSI, tissue saturation index.

### 3.3 Cognitive function

**FIGURE 4:**
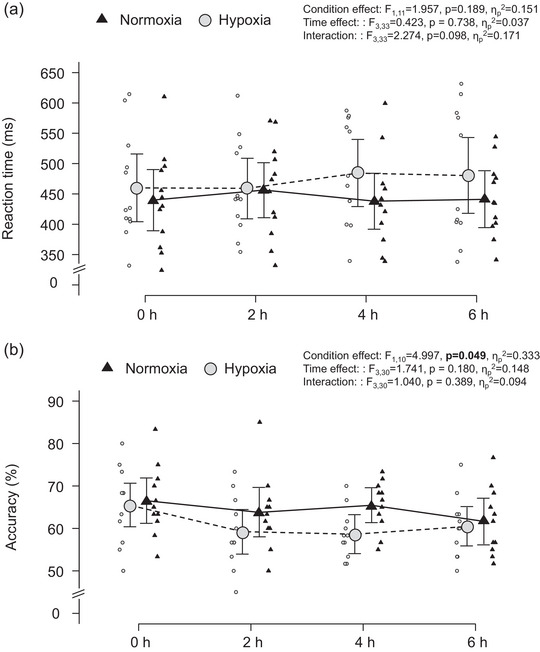
(a) Reaction time in the Go trial (Go/No‐Go task). (b) Accuracy of the memory task (as a percentage). Black triangles represent normoxia. Grey circles represent hypoxia. Small triangles/circles represent individual data.

Figure 4a illustrates RT in the Go/No‐Go task. Hypoxia did not affect RT (*P* = 0.189 vs. normoxia, main effect), and RT did not change as a function of time (*P* = 0.738, main effect). Accuracy of the Go/No‐Go task remained high throughout the experiment (Table 1) and was unaffected by hypoxia (*P* = 0.201 vs. normoxia, main effect) or time (*P* = 0.425, main effect). Figure 4b shows the accuracy of the memory task. Hypoxia impaired the accuracy of the memory task (*P* = 0.049 vs. normoxia, main effect), whereas time had no effect (*P* = 0.180, main effect).

## 4 DISCUSSION

The present study has identified three findings that have integrated translational relevance, albeit selectively constrained to male participants only. First, acute hypoxia was consistently associated with a reduction in both MCA (anterior) and PCA (posterior) DO_2_. Second, memory was generally impaired in hypoxia, and this was accompanied by a selective reduction in PCA DO_2_. Third, in stark contrast, central executive function remained preserved in hypoxia despite a comparable reduction in CDO_2_. Collectively, these findings suggest that a local reduction in perfusion and CDO_2_ to the posterior circulation selectively impair memory, whereas selective attention and response inhibition remain well preserved in hypoxia.

### 4.1 Memory

We observed impairments in memory during hypoxia, which is in line with previous studies (Lefferts et al., 2016b; Wang et al., 2013). In the memory task, participants were instructed to remember words during memorization, which required temporary storage until recall was performed. Memories initially require rapid synaptic plasticity within the hippocampus for formation and are gradually consolidated in neocortical networks (Kitamura et al., 2017; Kumaran et al., 2016). Therefore, the hippocampus is likely to play a crucial role in memory in the present study.

Rodent studies indicate that the hippocampus is more vulnerable to hypoxia‐mediated oxidative stress and subsequent hypoxic–ischaemic injury compared with the cortex (Hota et al., 2007; Maiti et al., 2006). Accordingly, the number of damaged cells in the CA3 region of the hippocampus has been shown to increase after exposure to 6400 m for 4 days (Shukitt‐Hale et al., 1996). Furthermore, hippocampal vasculature (i.e., lower capillary density and red blood cell velocity) might constrain cerebrovascular substrate (O_2_/glucose) delivery and account enhancing vulnerability to hypoxia (Shaw et al., 2021).

In the present study, hypoxia reduced CDO_2_, which coincided with the observed impairment in memory. The hippocampal blood supply is generally provided by the collateral branches of the PCA and the anterior choroidal artery (Perosa et al., 2020; Spallazzi et al., 2019), and therefore, the present results indirectly, albeit intuitively, suggest that O_2_ delivery to the hippocampus might be linked to cognitive dysfunction in hypoxia. In addition, we observed that Δoxy‐Hb and ΔTSI were selectively elevated during recall compared with memorization and during the Go/No‐Go task. Recall is involved in the activation of multiple regions, including the prefrontal cortex (Scalici et al., 2017), which is supported by our observations of greater increases in oxy‐Hb and TSI. These findings suggest that, in conjunction with the hippocampus, the prefrontal cortex might also be associated with memory recall. Hence, reductions in CDO_2_ in hypoxia might attenuate the activations in the prefrontal cortex during recall, contributing, at least in part, to the observed impairments in memory.

### 4.2 Central executive function

We demonstrated that RT in the Go/No‐Go task was unaffected by hypoxia despite a clear reduction in anterior (MCA) DO_2_. The MCA supplies the majority of the lateral surface of the hemisphere, including the prefrontal, motor, parietal and temporal cortices (Berman et al., 1984). These findings suggest that central executive function, as assessed in the present study, was either resistant to a reduction in MCA DO_2_ or, alternatively, that a threshold impairment in bioenergetic function needs to be surpassed before cognitive impairment ensues. However, it should be noted that the type of cognitive task has interpretive implications (Ando et al., 2020). The Go/No‐Go task requires selective attention and also control of motor inhibition (Guarino et al., 2018). Given the high accuracy of the performance and the absence of an impairment in hypoxia, this might be associated with relatively low task difficulty. Future studies using multiple central executive tasks (e.g., Stroop task) are encouraged to establish a wider understanding of the effect of hypoxia on central executive function.

### 4.3 Cerebral bioenergetics

The brain is a high‐flow organ that depends on acute increases in regional blood flow and DO_2_ to support increases in neural activity and synaptic transmission (Attwell et al., 2010; Bailey, 2019a, 2019b; Iadecola, 2004). Neurovascular coupling (NVC) refers to the mechanism that links neural activity to consequent increases in local CBF (Hendrikx et al., 2019). In the present study, cognitive activity failed to effect adequate increases in posterior perfusion and consequent CDO_2_ during the memory task in hypoxia. A recent rodent study demonstrated that NVC is weaker in the hippocampus compared with the visual cortex because of differences in vascular properties (Shaw et al., 2021). Thus, the present results would underscore the importance of basal CDO_2_ in the hippocampus for the maintenance/preservation of memory in hypoxia.

Equally, previous studies have documented that hypocapnia attenuates NVC (Caldwell et al., 2021; Szabo et al., 2011). In the present study, however, hypocapnia did not affect haemodynamic and CDO_2_ responses to cognitive activity. In these previous studies (Caldwell et al., 2021; Szabo et al., 2011), NVC was evaluated using visual stimulation. Visual stimulation generally evokes a robust NVC response that increases perfusion of the visual cortex substantially (Phillips et al., 2016), and the degree of NVC is likely to be greater (e.g., 9 ± 4 cm/s, peak absolute increase in PCAv during visual stimulation in the study by Caldwell et al., 2021) than that incurred in the present study. Hence, the absence of hypocapnic effects on haemodynamic and CDO_2_ responses to cognitive activity is probably attributable to the differences in the tasks (i.e., visual stimulation vs. cognitive task).

Blood glucose is the primary energy source for the brain (Gold, 1995). In the present study, hypoxia did not affect glucose and insulin concentrations, and as anticipated, blood lactate was mildly elevated in hypoxia. Hashimoto et al. (2018) have suggested that the increase in lactate production from extracerebral tissue is likely to support brain function. However, these increases were minimal and cognitive function did not improve. Thus, we can assume that changes in these substrates contribute little (if at all) to cognitive function.

### 4.4 Temporal kinetics

Reductions in CBF in the brain regions associated with memory were found to be more pronounced after prolonged (10 h) compared with acute (2 h) hypoxic exposure (Lawley et al., 2017), which suggests that prolonged hypoxia might reduce regional cerebral metabolism in the brain regions related to memory. However, in contrast to our original hypothesis, we found that memory did not become progressively worse in hypoxia, despite changes in CDO_2_; differences that might relate to the comparatively shorter exposure time used in the present study.

### 4.5 Experimental limitations

There are several major limitations that warrant critical consideration. First, our findings are constrained to male participants only, which is unfortunate given that incidence rates of neurogenerative diseases, notably Alzheimer's disease, are higher in women compared with men (Beam et al., 2018). Future researchers are actively encouraged to include both men and women to help identify biological sex differences in responses to various stimuli that could be influential for brain health and brain ageing. Second, despite retrospective analyses revealing that we were adequately powered (1 − β = ≥ 0.80 at *P* < 0.05) to detect main effects for all primary outcomes, we recognize that our sample size is small and that this is likely to have contributed to the absence of hypoxic effects on central executive function. Given that we were not in a position to include more participants owing to logistical/financial constraints, future research needs to consider larger sample sizes to draw firmer conclusions and make these findings more applicable to the general population. Third, MCAv and PCAv were measured using TCD, reflecting an indirect surrogate measure of regional CBF. In hypoxia, the net CBF response reflects a balance between hypoxic cerebral vasodilatation and hypocapnic vasoconstriction driven by hyperventilation‐induced hypocapnia (Ogoh et al., 2014). Given that MCA diameter appears to increase in hypoxia (Wilson et al., 2011), we cannot rule out the possibility that we underestimated volumetric changes in hypoxia. In addition, hippocampal vascularization seems to be categorized into a mixed supply from both the PCA and the anterior choroidal artery (Perosa et al., 2020). This implies that there are inter‐individual differences in hippocampal blood flow supply. Hence, associations between cognitive function and regional cerebral perfusion/CDO_2_ warrant further investigation. Follow‐up studies using advanced imaging techniques, such as arterial spin labelling, would be helpful to improve our understanding of the association between memory and regional CBF and substrate delivery in hypoxia. Finally, the extent to which hypobaria per se (and not hypoxia) impacts cognitive function remains to be established, despite preliminary evidence for comparable effects (Hohenauer et al., 2022).

## 5 CONCLUSIONS

The present study examined the effects of acute hypoxia (∼7 h) on central executive (Go/No‐Go) and non‐executive (memory) function. In hypoxia, cerebral bioenergetic function taking the form of MCA and PCA DO_2_ were consistently lower compared with normoxia. The reduction in PCA DO_2_ was accompanied by impaired memory, whereas selective attention and response inhibition remained well preserved, despite comparable reductions in CDO_2_. Collectively, these findings, albeit selectively constrained to males, suggest that cognitive function, in particular memory, is selectively vulnerable in hypoxia.

## AUTHOR CONTRIBUTIONS

All experiments were conducted in the Neurovascular Research Laboratory at University of South Wales. Damian M. Bailey obtained funding. Soichi Ando, Hayato Tsukamoto, Benjamin S. Stacey, Shigehiko Ogoh and Damian M. Bailey conceived and designed the research. All authors contributed to the acquisition, analysis and interpretation of data. Soichi Ando, Shigehiko Ogoh and Damian M. Bailey drafted the manuscript, and all authors revised it critically and contributed intellectual content. All authors approved the final version of the manuscript, agree to be accountable for all aspects of the work and will ensure that any questions concerning the accuracy or integrity of any part of this work are appropriately investigated and resolved. All persons designated as authors qualify for authorship, and all those who qualify for authorship are listed.

## CONFLICT OF INTEREST

Damian M. Bailey is Editor‐in‐Chief of *Experimental Physiology*, Chair of the Life Sciences Working Group and a member of the Human Spaceflight and Exploration Science Advisory Committee to the European Space Agency and is a member of the Space Exploration Advisory Committee to the UK Space Agency. Damian M. Bailey is affiliated to the companies FloTBI, BrainEx and OrgEx, focused on the technological development of novel biomarkers of brain injury in humans.

## Data Availability

Original data arising from this research are available directly from Professor Damian Miles Bailey upon reasonable request.
